# Mast Cells Respond to *Candida albicans* Infections and Modulate Macrophages Phagocytosis of the Fungus

**DOI:** 10.3389/fimmu.2018.02829

**Published:** 2018-11-30

**Authors:** Marco De Zuani, Giuseppe Paolicelli, Teresa Zelante, Giorgia Renga, Luigina Romani, Alessandra Arzese, Carlo E. M. Pucillo, Barbara Frossi

**Affiliations:** ^1^International Clinical Research Center, St. Anne's University Hospital Brno, Brno, Czechia; ^2^Department of Medicine, University of Udine, Udine, Italy; ^3^Department of Experimental Medicine, University of Perugia, Perugia, Italy

**Keywords:** mast cells, *candida*, macrophages, phagocytosis, microbiota

## Abstract

Mast cells (MCs) are long-lived immune cells widely distributed at mucosal surfaces and are among the first immune cell type that can get in contact with the external environment. This study aims to unravel the mechanisms of reciprocal influence between mucosal MCs and *Candida albicans* as commensal/opportunistic pathogen species in humans. Stimulation of bone marrow-derived mast cells (BMMCs) with live forms of *C. albicans* induced the release of TNF-α, IL-6, IL-13, and IL-4. Quite interestingly, BMMCs were able to engulf *C. albicans* hyphae, rearranging their α-tubulin cytoskeleton and accumulating LAMP1^+^ vesicles at the phagocytic synapse with the fungus. *Candida*-infected MCs increased macrophage crawling ability and promoted their chemotaxis against the infection. On the other side, resting MCs inhibited macrophage phagocytosis of *C. albicans* in a contact-dependent manner. Taken together, these results indicate that MCs play a key role in the maintenance of the equilibrium between the host and the commensal fungus *C. albicans*, limiting pathological fungal growth and modulating the response of resident macrophages during infections.

## Introduction

Mast cells (MCs) are immune cells belonging to the innate arm of immunity. They originate in the bone marrow from a hematopoietic progenitor through the myeloid lineage but, unlike other myeloid-derived cells, MCs progenitors leave the bone marrow at an early stage of differentiation to enter the circulation. Once in the bloodstream, they rapidly migrate to the periphery and complete their differentiation into mature MCs with tissue-specific phenotypes (1). These cells mainly localize at mucosal sites and are found in close contact with epithelial cells and venules. They differentially express a wide plethora of pathogen recognition receptors (PRRs), cytokines and chemokine receptors, as well as costimulatory molecules by virtue of their tissue-specificity. Moreover, triggering of MCs by specific stimuli results in their activation and in the release of different pre-stored and *de novo* synthesized mediators (2, 3). Albeit being relegated to mere effectors of allergic processes for many years, MCs are now believed to be important tissue-resident sentinels and have been described to interact with the host microbiota (4, 5).

*Candida* spp. are commensal fungi that colonize mucous membranes and the skin of healthy individuals and among all the species, *Candida albicans* is the most common in human mycobiota. However, they can cause severe invasive diseases in patients hospitalized in intensive care units, with solid tumors or hematological malignancy, undergoing surgery or being treated with broad-spectrum antibiotics. Albeit being only poorly considered as a public health concern, over 800 million people worldwide suffer from life-threatening fungal-related diseases and it is estimated that *C. albicans* is responsible for more than one half of the cases of candidaemia, which mortality rates in Europe vary between 28 and 59% (6, 7).

Among *Candida* species, *C. albicans* is the only one able to grow as a unicellular yeast and as a filamentous hyphal and pseudohyphal forms (8). This property is rather important as *C. albicans* hyphal growth is an important virulence factor and represent a key step for tissue invasion processes (9). The immune response to *C. albicans* begins with the recognition of specific pathogen-associated molecular patterns (PAMPs) by the innate arm of the immune system. The recognition of fungal PAMPs is mediated by several PRRs, including C-type lectin receptors (CLRs), Toll-like receptors (TLRs), and intracellular NOD-like receptors (NLRs) (10). Dectin-1 is the best characterized CLR and is fundamental for the recognition of β-glucans and subsequent production of pro- and anti-inflammatory cytokines. On the other side, TLR2, TLR4, and TLR6 are the main TLRs that are involved in the recognition of the fungal cell wall mannoproteins and can cooperate with dectin-1 to boost cytokine expression in response to β-glucans (11, 12).

Despite their potential role during fungal infections, interactions between MCs and fungi have been only poorly investigated and published data are often contradictory. Gastrointestinal colonization with *C. albicans* induced MCs infiltration and degranulation, increasing the permeability of the gastrointestinal mucosa (13). Rat peritoneal MCs as well as murine bone marrow-derived MCs (BMMCs) were shown to be able to phagocytose heat killed and opsonized live *C. albicans* yeasts and to produce nitric oxide (but not ROS) in a mechanism involving both TLR2 and dectin-1 (14, 15). On the contrary, a recent study demonstrated that BMMCs released ROS and several pro-inflammatory cytokines in response to *in vitro* stimulation with *C. albicans* yeasts and hyphae (16). *Candida* challenge also induced degranulation of human MCs and the release of pro- and anti-inflammatory cytokines as well as of tryptase-containing MC-extracellular traps (17).

Our work adds new tiles on the big picture of MCs role during fungal infection, describing the tight interaction between these cells and *C. albicans*, as well as their control of macrophages activation and fungal clearance.

## Materials and methods

### Mice

C57BL/6 mice were purchased from Envigo (The Netherlands) and maintained at the animal facility of the Department of Medicine, University of Udine (Italy). Dectin-1^−/−^ femurs and tibiae were kindly gifted by Prof. Gordon Brown, University of Aberdeen (Aberdeen, UK). All animal experiments were approved by the OPA (Organismo per il Benessere Animale) of the local committee in accordance with institutional guidelines and National law (D.Lgs. 26/2014).

### BMMCs generation

Bone marrow-derived mast cells (BMMCs) were obtained from 6- to 8- weeks-old mice by *in vitro* differentiation of bone marrow-derived progenitors obtained from mice femurs and tibiae. Precursor cells were cultivated in complete–IL-3 medium [RPMI 1,640 medium (Euroclone) supplemented with 20% FBS (Sigma Aldrich), 100 U·ml^−1^ penicillin, 100 μg·ml^−1^ Streptomycin, 2 mM Glutamine, 20 mM Hepes, non-essential amino acid, 1 mM Sodium Pyruvate (Euroclone), 50 mM β-mercaptoethanol (Sigma Aldrich), and 20 ng·ml^−1^ IL-3 (Peprotech)] at 37°C in 5% CO_2_ atmosphere. After 5 weeks, BMMCs differentiation was confirmed by flow cytometry by staining with fluorochrome-conjugated anti-FcεRIa (MAR-1) and anti-cKit (ACK2) antibodies (eBiosciences). BMMCs were usually ≥96% cKit^+^ and FcεRIa^+^.

### BMMCs activation

Before all *in vitro* experiments, BMMCs were starved for 1 h in IL-3–free complete RPMI medium [RPMI 1,640 medium (Euroclone) supplemented with 10% FBS (Sigma), 100 U·ml^−1^ penicillin, 100 μg·ml^−1^ Streptomycin, 2 mM Glutamine, 20 mM Hepes, non-essential amino acid, 1 mM Sodium Pyruvate (Euroclone), and 50 mM β-mercaptoethanol (Sigma Aldrich)].

For IgE-dependent activation, BMMC were sensitized in IL-3–free complete RPMI medium for 3 h with 1 μg·ml^−1^ of dinitrophenol (DNP)-specific IgE, washed twice and challenged with 100 ng·ml^−1^ DNP (Sigma- Aldrich).

For *C. albicans* infections, 10^6^ BMMCs were stimulated with *C. albicans* yeast (1:1 ratio) or hyphae (1:10 ratio) at a final concentration of 2·10^6^ cells·ml^−1^ in IL-3–free complete RPMI medium. In order to limit fungal growth, amphotericin-B (Sigma Aldrich) was added to each well at a final concentration of 10 ng·ml^−1^. RNA extraction was performed before the addition of amphotericin-B.

### BMMCs phagocytosis of *C. albicans*

Phagocytosis of *C. albicans* by BMMCs was assessed by flow cytometry. *C. albicans* yeasts were labeled for 20 min with 5 μM Cell Proliferation Dye eFluor 670 (CPD, eBioscience) at 37°C in 5% CO_2_ atmosphere, following manufacturer's instructions. CPD-labeled *C. albicans* was seeded to BMMCs at a 10:1 ratio on 24-well plates in IL-3–free complete RPMI medium and incubated for 90 min at 37°C in 5% CO_2_ atmosphere. As a negative control of phagocytosis, some BMMCs were pretreated with 10 μM cytochalasin-D 1 h before the addition of CPD-labeled *C. albicans* yeasts. Alternatively, the phagocytosis was performed at 4°C to block active endocytosis processes. Cells were then harvested, stained for cKit and acquired. Phagocyting BMMCs were determined as cKit^+^ CPD^+^ double-positive cells.

### BMMCs degranulation assay

BMMCs degranulation response was determined as the percentage of β-hexosaminidase released. 0.5·10^6^ BMMCs were incubated in Tyrode's buffer (10 mM HEPES buffer [pH 7.4], 130 mM NaCl, 5 mM KCl, 1.4 mM CaCl2, 1 mM MgCl2, 5.6 mM glucose, and 0.1% BSA) with or without the addition of 10% FBS and stimulated with the same number of *C. albicans* yeasts at 37°C for the indicated time points. As a positive control, 0.5·10^6^ BMMCs were sensitized in complete RPMI medium for 3 h with 1 μg·ml^−1^ of dinitrophenol (DNP)-specific IgE, then washed twice, resuspended in Tyrode's buffer and challenged with 100 ng·ml^−1^ DNP (Sigma-Aldrich). The enzymatic activity of the released β-hexosaminidase was assessed by the cleavage of its synthetic substrate (p-nitrophenyl N-acetyl- glucosamide, Sigma Aldrich) in p-nitrophenol and measuring the p-nitrophenol absorbance at 405 nm with a plate spectrophotometer. Results are expressed as the percentage of β-hexosaminidase released over β-hexosaminidase retained in the cytoplasm. Leukotrienes C4, D4, and E4 were measured in the same samples using a specific detection kit (GE Healthcare) according to the manufacturer's instructions.

### Purification of peritoneal macrophages

The peritoneum of 8- to 12-weeks-old C57BL/6 mice was lavaged using a PBS solution containing 100 U·ml^−1^ penicillin and 100 μg·ml^−1^ Streptomycin (Euroclone). Following lavage, the cells were washed, resuspended in complete RPMI medium, plated in 24-well plates at a concentration of 0.5·10^6^ cells/well, and cultured for 6 h at 37°C in a 5% CO_2_ atmosphere. Non-adherent cells were removed by washing the cells twice with PBS, and adherent cells were cultured overnight in complete RPMI at 37°C in a 5% CO_2_ atmosphere. Peritoneal macrophages purity was confirmed by flow cytometry and immunofluorescence by staining with fluorochrome-conjugated anti-F4/80 (BM8), anti-CD11b (M1/70) and anti-MHC-II (M5/114.15.2) antibodies (BioLegend).

### *Candida albicans* cultures

Wild-type *C. albicans* SC5314 strain yeasts were seeded on BBL Sabouraud Dextrose Agar (Becton Dickinson and Company) supplemented with 50 μg·ml^−1^ chloramphenicol and incubated at 30°C for 24 h. To generate *C. albicans* hyphae, 10^7^ yeast cells were resuspended in complete RPMI medium, seeded into T-25 adhesion flasks and allowed to germinate for 3 h at 37°C. Hyphae were harvested by scraping, centrifuged at 700 × g for 10 min and washed with PBS.

### Flow cytometry

0.5·10^6^ cells were harvested, washed with PBS and stained for 30 min at 4°C in the dark with fluorochrome-conjugated monoclonal antibodies. Cells were then washed twice with PBS and acquired with a FACSCalibur flow cytometer (Becton Dickinson). Data were analyzed with FlowJo software (FlowJo LLC).

### Time-lapse bright-field microscopy

MC-*C. albicans* interaction was analyzed by time-lapse epiluminescent microscopy using the Leica AF6000LX system (DMI6000-B microscope equipped with a DFC350FX camera) at a magnification of 40 × . Before each experiment, BMMCs were labeled with FAST DiI (Invitrogen) according to the manufacturer's instructions. 0.5·10^6^ BMMCs and 0.5·10^6^ *C. albicans* yeasts (ratio 1:1) were plated on 8-well PermanoxR Chamber Slide (Lab-Tek, Nunc). The chamber was placed at 37°C in 5% CO_2_ atmosphere. Phase contrast images were recorded every 10 min for a total of 12 h and resulting video-recorded movies were processed with LAS AF (Leica) and Fiji (ImageJ) software (18).

### Immunofluorescence

Cells were seeded onto sterile glass coverslip in a 24 well plate and stimulated. Non-adherent cells were gently removed by pipetting, coverslips washed with PBS and fixed for 20 min with 4% formaldehyde (Sigma Aldrich). Cells were permeabilized for 5 min with 0.1% Triton-X100 (Fluka) and incubated for 30 min with PBS + 10% FBS (Sigma Aldrich) to reduce unspecific binding. Coverslips were incubated with primary antibodies (diluted in PBS + 5% FBS) overnight at 4°C in a humidified chamber. AlexaFluor−488, −546, or −647 conjugated anti-mouse, anti-rabbit or anti-rat secondary antibodies (Invitrogen) were incubated for 1 h at room temperature. Coverslips were washed and mounted on SuperFrost glass slides (Menzel) with Mowiol 40–88 mounting media containing 2.5% 1,4-diazobicyclo-[2,2,2]-octane (Sigma Aldrich). Images were acquired with a with a Leica DM IRBE microscope equipped with a TCS-SP confocal scanner head (with 488 nm Ar and 543–633 nm HeNe lasers) at a magnification of 63 × and processed with Fiji (ImageJ) software (18). Antibodies (clones and vendors where applicable): α-tubulin (DM1A, Sigma Aldrich), Candida (Abnova), CD11b (M1/70, Thermo Fisher), F4/80 (BM8, BioLegend), FcεR β chain (JRK b), LAMP-1 (1D4B, eBiosciences), TBP (SI-1, Santa Cruz).

### Cytokine ELISA assays

Supernatants for cytokine quantitation were collected 3 or 24 h after BMMCs stimulation. Supernatants were assessed for TNF-α, IL-6, IL-13, and IL-4 using specific ELISA kits (eBiosciences) according to manufacturer's instructions.

### Macrophage chemotaxis and migration assay

Chemotaxis of peritoneal macrophages was evaluated using the ibidi® μ-Slide Chemotaxis kit according to the manufacturer's instructions. ≈15,000 peritoneal macrophages were seeded in the observation area and the slide incubated at 37°C in 5% CO_2_ atmosphere. After cell attachment, non-adherent cells were removed by washing three times with PBS. Twenty four hours after cell seeding, reservoirs were filled with either complete RPMI medium (10% FBS) or conditioned media and the slides were immediately placed at 37°C in 5% CO_2_ atmosphere. DIC images were recorded at 10 × magnification every 10 min for a total of 24 h and resulting video-recorded movies were processed with LAS AF software (Leica). At least 25 cells per condition were manually tracked with Fiji Software (ImageJ) and resulting data were analyzed with the Chemotaxis and Migration Tool software (ibidi) (18).

Macrophage chemotaxis during live *C. albicans* infection was assessed using 8 μm Transwell® inserts (Corning). Briefly, 10^5^ peritoneal macrophages were seeded in serum-free media in the upper chamber of a 24 well-Transwell® system. The lower chamber was filled with serum-free media containing or not: conditioned media from *C. albicans* alone, BMMCs alone or *C. albicans*-infected BMMCs; 2·10^6^ ml^−1^ BMMCs, 2·10^6^ ml^−1^ *C. albicans* yeasts or 2·10^6^ ml^−1^ BMMCs stimulated with *C. albicans* yeast (1:1 ratio); 100 ng ml^−1^ MCP-1. Chemotaxis was allowed overnight, then inserts were collected, carefully washed and stained with crystal violet (0.5% in 25% methanol) for 10 min. Migrated cells were counted in 3 random fields and the percentage of migration was calculated on the total number of seeded macrophages.

### Macrophage phagocytosis assay

*Candida albicans* yeasts were labeled for 20 min with 5 μM Cell Proliferation Dye eFluor 670 (CPD, eBioscience) at 37°C in 5% CO_2_ atmosphere, following manufacturer's instructions. CPD-labeled *C. albicans* was seeded to BMMCs at a 1:1 ratio or plated alone on 24-well plates, incubated for 3 h at 37°C in 5% CO_2_ atmosphere and harvested by scraping. Peritoneal macrophages received BMMCs co-cultured with *C. albicans*, naïve BMMCs, and CPD-labeled *C. albicans* or CPD-labeled *C. albicans* alone at a 1:1:1 ratio. As a negative control of phagocytosis, some macrophages were pretreated with 1 μM cytochalasin-D for 1 h. After 1 h, cells were harvested by scraping, washed with PBS and stained with anti-F4/80 (BM8, Biolegend). Flow cytometry was used to quantify the number of F4/80^+^ cells that had engulfed CPD-labeled *C. albicans*. Percentage of phagocytosis was calculated by subtracting the percentage of double positive cells in presence of cytochalasin-D to the percentage of double positive cells in non-treated macrophages. Phagocytosis index was further determined as fold-change over the phagocytosis percentage of macrophages stimulated with CPD-labeled *C. albicans* alone. In some experiment, 50 pg·ml^−1^ recombinant IL-4 (Peprotech), 100 pg·ml^−1^ recombinant TNF-α (Immunotools), 10 μg·ml^−1^ anti-IL-4 neutralizing antibody (11B11, eBioscience), 10 μg·ml^−1^ anti-TNF-α neutralizing antibody (MP6-XT22, Miltenyi Biotec), or conditioned media were used to stimulate peritoneal macrophages together with BMMCs and *C. albicans*.

### RNA extraction and real-time PCR analyses

Cells were lysed with EURO GOLD TriFast (Euroclone) and total RNA extracted with the phenol-chloroform protocol according to manufacturer's instructions. Total RNA was quantified using a NanoDrop^TM^ spectrophotometer (ThermoFischer) and retro-transcribed with the SensiFAST™ cDNA Synthesis kit (Bioline). Quantitative qPCR analyses were performed with SYBR Green chemistry (BioRad) using a BioRad iQ5 real-time PCR detection systems. Target genes expression were quantified with the ΔΔCt method using g3pdh (glyceraldehyde 3-phosphate dehydrogenase) as normalizer gene. PCR primers used are as follows: *Tnf*α (5′-AGGCACTCCCCCAAAAGATG-3′ and 5′-CCATTTGGGAACTTCTCATCCC-3′), *il4* (5′-AGCCATATCCACGGATGCGACAAA-3′ and 5′-AATATGCGAAGCACCTTGGAAGCC-3′), *il6* (5′-ACCACTTCACAAGTCGAAGGCTTA-3′ and 5′-TCTGCAAGTGCATCATCGTTGTTC-3′), *il13* (5′-AGGAGCTTATTGAGGAGCTGAGCA-3′ and 5′-TGGAGATGTTGGTCAGGGAATCCA-3′), *g3pdh* (5′-TCAACAGCAACTCCCACTCTTCCA-3′ and 5′-ACCCTGTTGCTGTAGCCGTATTCA-3′).

### Statistical analyses

Unless otherwise indicated, results are expressed as mean (SD) of at least three independent experiments. Data were analyzed using ordinary one-way ANOVA, paired two-way ANOVA, Student's *t*-test or Kruskal-Wallis tests (GraphPad Prism v6). Rayleigh tests were performed with the Chemotaxis and Migration Tool software (ibidi). A confidence level of 95% was used. ^*^p < 0.05, ^**^p < 0.01, ^***^p < 0.001, ^****^p < 0.0001.

## Results

### MC—immunological synapse

**Figure 1:**
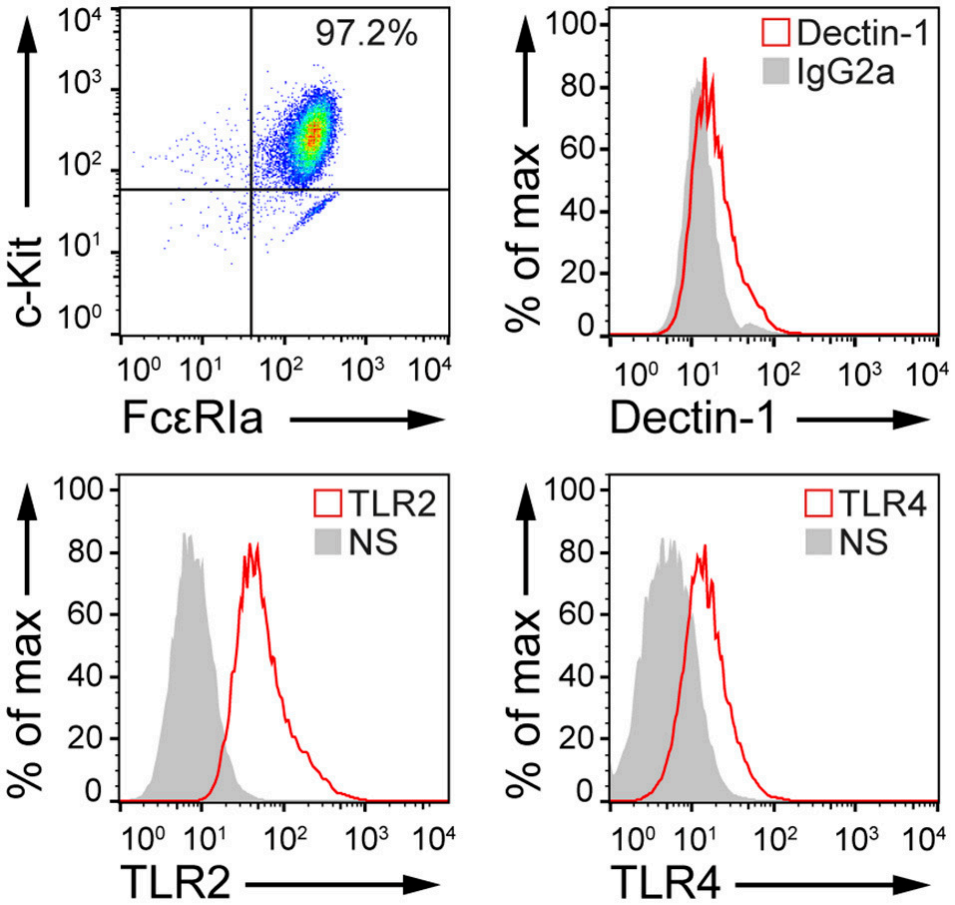
MCs express PRRs involved in microbial recognition. FACS analyses of cKit^+^, FcεRIa^+^ BMMCs indicate that these cells express dectin-1, TLR2, and TLR4. NS, non-stained.

**Figure 2:**
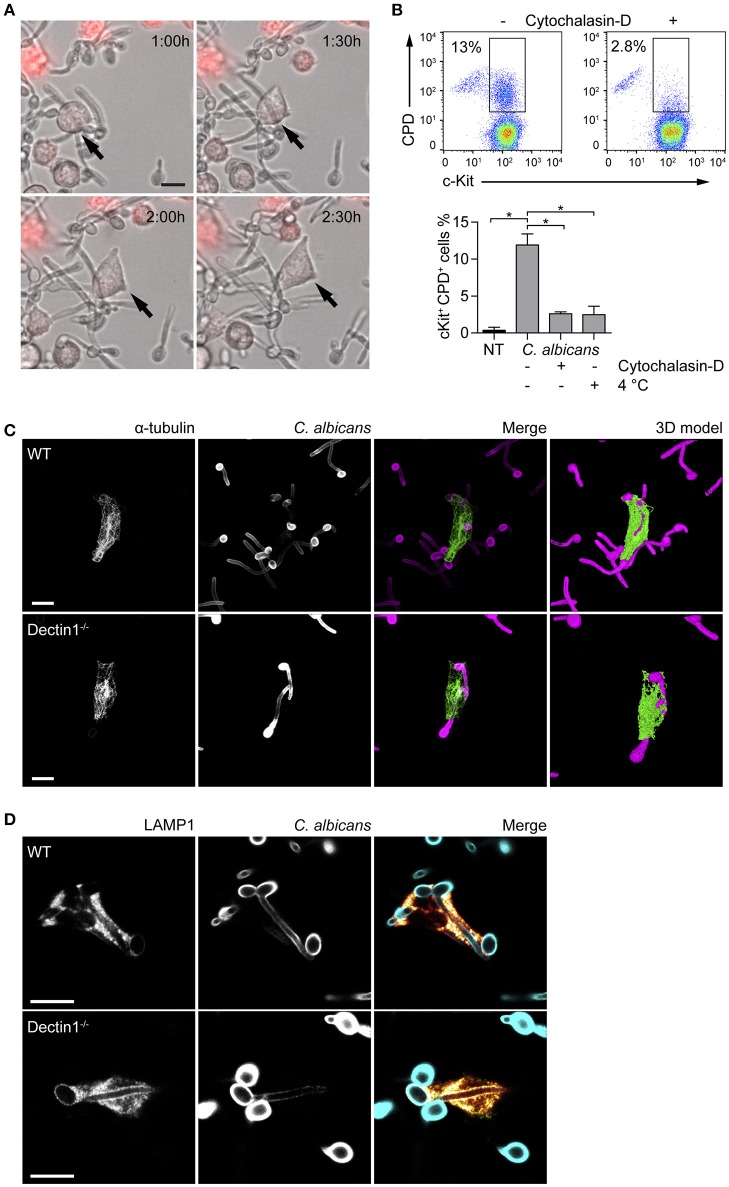
MCs tightly interact with *C. albicans* hyphae. **(A)** Time-lapse microscopy show BMMCs (stained in red) intimately interacting with *C. albicans* hyphae after 90 min of co-culture, resembling the phenotype of “frustrated phagocytosis.” On the contrary, phagocytosis of yeasts was not observed. **(B)** MCs engulfment of *C. albicans* was assessed by flow cytometry after 90 min of co-culture between BMMCs and CPD-labeled *C. albicans* yeasts in the presence or absence of cytochalasin-D or at 4°C. Representative dot plot and mean ± SD of three independent experiments are shown. Data were analyzed with paired one-way ANOVA and Tukey's multiple comparison test. **(C**,**D)** Immunofluorescence images show that BMMCs rearrange their α-tubulin cytoskeleton during the interaction and accumulate LAMP1^+^ vesicles at the interface with the fungus. *Dectin-1*^−/−^ BMMCs display an identical ability to interact with *C. albicans* hyphae. Scale bars: 10 μm. ^*^p < 0.05.

Fungal recognition by immune cells specifically relies on the recognition of fungal PAMPs by cellular PRRs (12). In order to assess whether BMMCs could recognize *C. albicans*, the expression level of different PRRs involved in fungal recognition was analyzed by flow cytometry. As reported in Figure 1, BMMCs expressed dectin-1 as well as TLR2 and TLR4. To better dissect the interaction between MCs and *C. albicans*, BMMCs were cocultured with live *C. albicans* both in the yeast and hyphal forms. Intriguingly, after a few hours of co-culture, MCs were found to tightly interact with the hyphal form of the fungus in a way that resembled phagocytosis. Time-lapse bright field microscopy experiments showed that MCs interacted with *C. albicans* as soon as it changed its morphology from yeasts to hyphae but not with yeasts alone, suggesting that this phenomenon specifically relies on the progression of *Candida* germination (Figure 2A and Supplementary Video 1). Flow cytometric analysis of BMMC-*C. albicans* shows that a considerable number of BMMCs are able to phagocytose the fungus (Figure 2B). Interestingly, this process was not a consequence of the fungal invasion of MCs but was rather mediated by MC's actin dynamics, as the addition of cytochalasin-D, a potent actin polymerization inhibitor, almost completely inhibited the process (Figure 2B).

Dectin-1 signaling is known to be activated only when the receptor binds to particulate β-glucans. This interaction induces the receptor to cluster in synapse-like structures (called “phagocytic synapses”) to which signaling molecules are recruited (19). Albeit *C. albicans* hyphae β-glucans are shielded by a layer of mannoproteins and thus fail to activate dectin-1, it has also been hypothesized that this receptor may be responsible for the recognition of hyphal β-glucans probably due to the presence of thinner mannan fibrils (20). Interaction with *C. albicans* by dectin-1^−/−^ BMMCs was comparable with WT BMMCs. Immunofluorescence staining showed that partial engulfment of *C. albicans* hyphae induced the rearrangement of the α-tubulin cytoskeleton in both WT and dectin-1^−/−^ BMMCs (Figure 2C). 3D-modeling of α-tubulin-stained BMMCs, indicate that BMMCs are able to “wrap” around the fungal hypha (Figure 2C), resembling the phenotype of the so-called frustrated phagocytosis (21, 22). In order to define whether this behavior could be ascribed as phagocytosis or not, BMMCs were stained for two markers of early- and late-endosomes. During phagosome maturation, phagosomes acquire different surface molecules (e.g., Rab GTPases) which play key roles in the process of maturation. The early-endosome antigen 1 (EEA1) is involved in the initial stages of the maturation process by binding to PIP3 and mediating endosomes fusion. On the other side, the late phase marker lysosomal-associated membrane protein 1 (LAMP1) is acquired at the late stages of maturation, after the endosomes have fused with acidic lysosomes (23). None of the cells stained for EEA1 (not shown) while most of them stained positively for LAMP1. Interestingly, both WT and dectin-1^−/−^ BMMCs stained positively for LAMP1, suggesting that this receptor is not required for the accumulation of LAMP1^+^ vesicles (Figure 2D).

### MCs degranulation in response to fungal challenge

**Figure 3:**
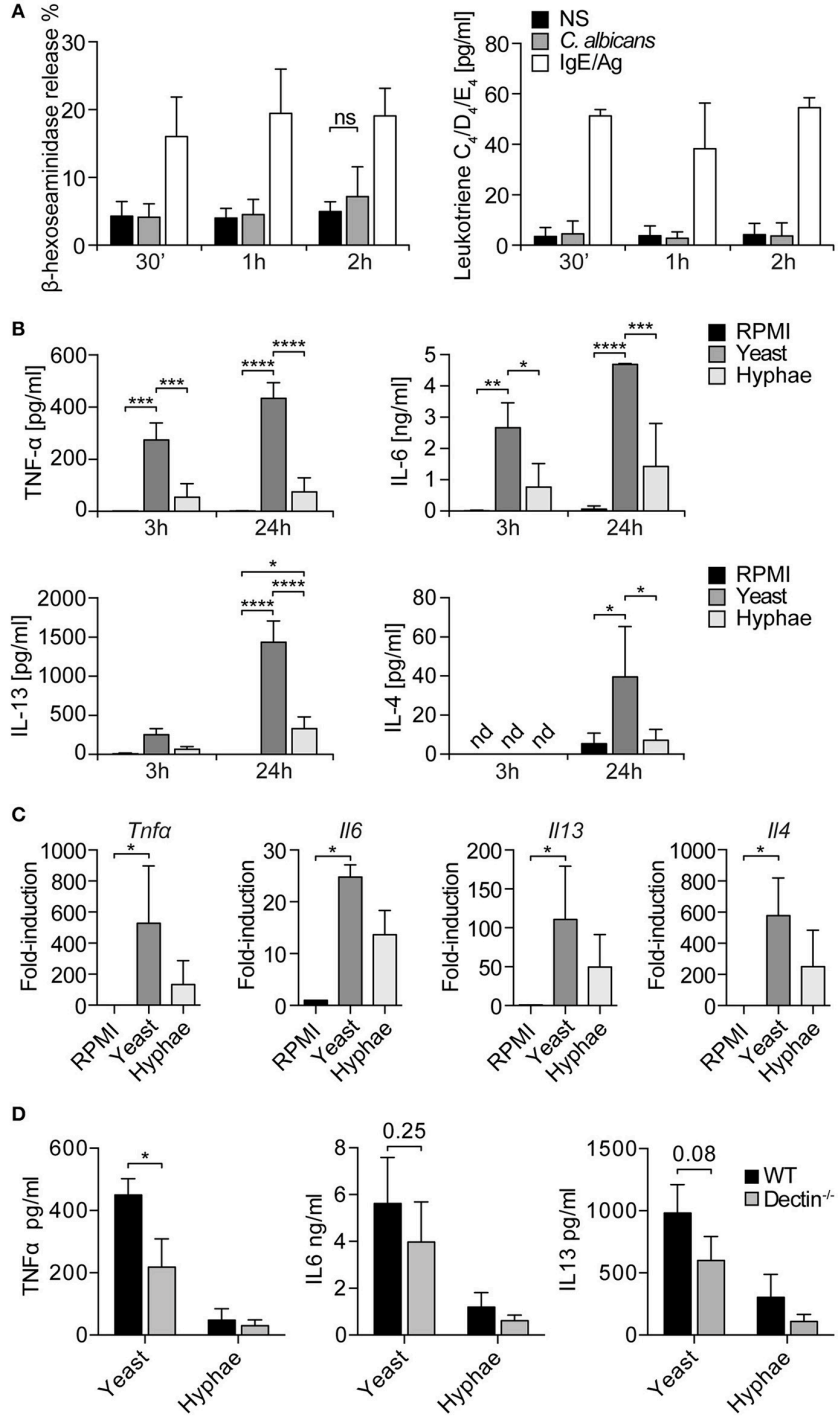
MCs degranulation and cytokine release in response to *C. albicans*. **(A)** BMMCs degranulation in response to *C. albicans* was evaluated by the release of β-hexosaminidase and the synthesis of leukotrienes. No degranulation was observed after 30 min, 1 or 2 h. Data were analyzed with paired 2-way ANOVA. NS, statistically non-significant. **(B)** Stimulation of BMMCs with *C. albicans* induced a quick release of TNF-α, IL6, and IL13 (already detectable after 3 h) and of IL-4. *C. albicans* yeasts induced a more prominent cytokine release compared to hyphae. Data were analyzed with paired 2-way ANOVA. **(C)** Expression levels of *tnf*α*, il6, il13, il4* were confirmed by qPCR after 3 h of co-culture. Data were analyzed by Kruskal Wallis test. Nd, non-detected. **(D)** Challenge of *dectin-1*^−/−^ MCs with *C. albicans* resulted in an impaired release of TNF-α, IL-6, and IL-13 compared to WT controls. Data were analyzed with paired 2-way ANOVA and Tukey's multiple comparison test. ^*^p < 0.05, ^**^p < 0.01, ^***^p < 0.001, ^****^p < 0.0001.

Seen that LAMP1 is also considered a marker of degranulation, MCs degranulation in response to *C. albicans* was evaluated. Fungal challenge was performed in the presence of 10% serum in order to allow *C. albicans* switch to the hyphal form, and the release of β-hexosaminidase and leukotrienes C4, D4, and E4 was determined after 30 min, 1, and 2 h. The release of β-hexosaminidase was minimally increased over the control only after 2 h of stimulation, while leukotrienes levels remained constant at all the time points (Figure 3A). IgE/Ag stimulation was used as positive control of MCs degranulation. Taken together, these data indicate that, in our setup, BMMCs do not degranulate in response to the encounter of *C. albicans*.

### Cytokine release in response to fungal challenge

MCs can release a broad range of *de novo* synthesized mediators which play an important role in the modulation of the immune response to pathogens (24). To understand the role of MC-derived mediators during fungal infections, MCs were co-cultured with live *C. albicans* yeast and hyphae and culture supernatants assessed for different cytokines. After 3 h of co-culture, it was already possible to detect IL-6, IL13, and TNF-α, while after 24 h also IL-4 was detected in culture supernatants (Figure 3B). Interestingly, *C. albicans* yeasts were more effective than hyphae in inducing cytokine release from MCs. These data were also confirmed by gene expression analyses that revealed a strong upregulation of *tnf-*α, *il6, il13*, and *il4* genes. Again, stimulation with *C. albicans* yeasts rather than hyphae induced higher levels of cytokines expression (Figure 3C). To assess whether dectin-1 played a role in MCs activation by *C. albicans*, dectin-1^−/−^ BMMCs were co-cultured with the fungus and cytokine levels were assessed after 24 h. Stimulation of *dectin-1*^−/−^ BMMCs with *C. albicans* yeast and hyphae resulted in an impaired release of TNF-α, IL-6, and IL-13 compared to WT controls, both during the stimulation with yeasts and hyphae (Figure 3D). Notably, cytokine release was only impaired and not completely abolished, in line with the hypothesis that dectin-1 is not the only receptor involved in *C. albicans* recognition (11).

### Macrophage crawling is increased in the presence of activated MCs

Clearance of fungal pathogens rely mostly on the activity of phagocytic cells and especially on neutrophils and macrophages, and depletion of mononuclear phagocytes has been described to worsen fungal proliferation and overall survival (25). MCs interact with many members of the innate and adaptive immune system and can affect monocyte/macrophage behavior during infections (26, 27). Thus, we aimed to determine how BMMCs could induce macrophage migration and modulate their ability to phagocyte *C. albicans*.

**Figure 4:**
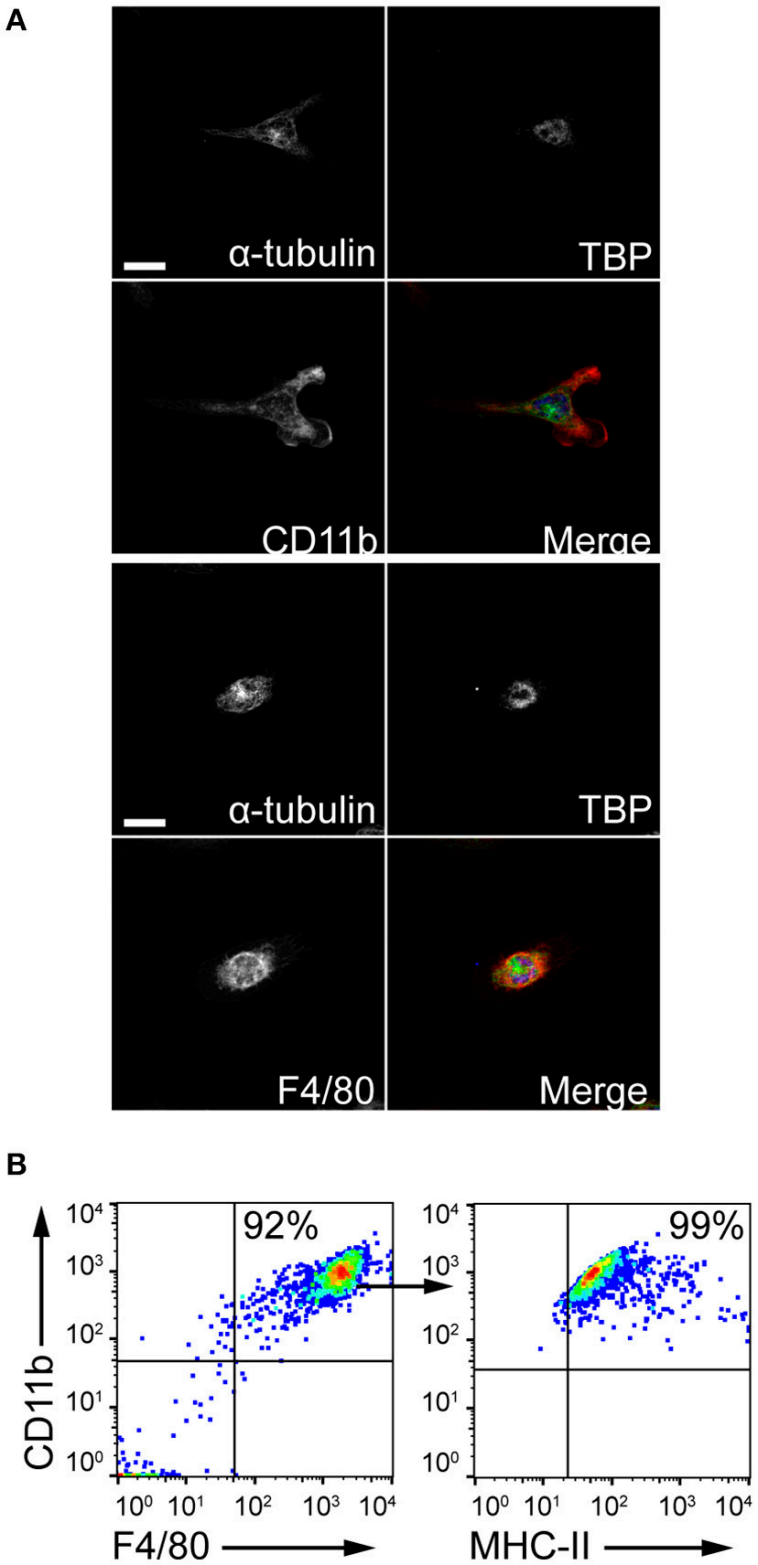
Peritoneal macrophages purification. Peritoneal macrophages were obtained from the peritoneal lavage of C57BL/6 mice and checked for purity. **(A)** Z-stack averaging of confocal images. Scale bars: 10 μm. **(B)** Flow cytometry gating strategy to confirm macrophages purification.

**Figure 5:**
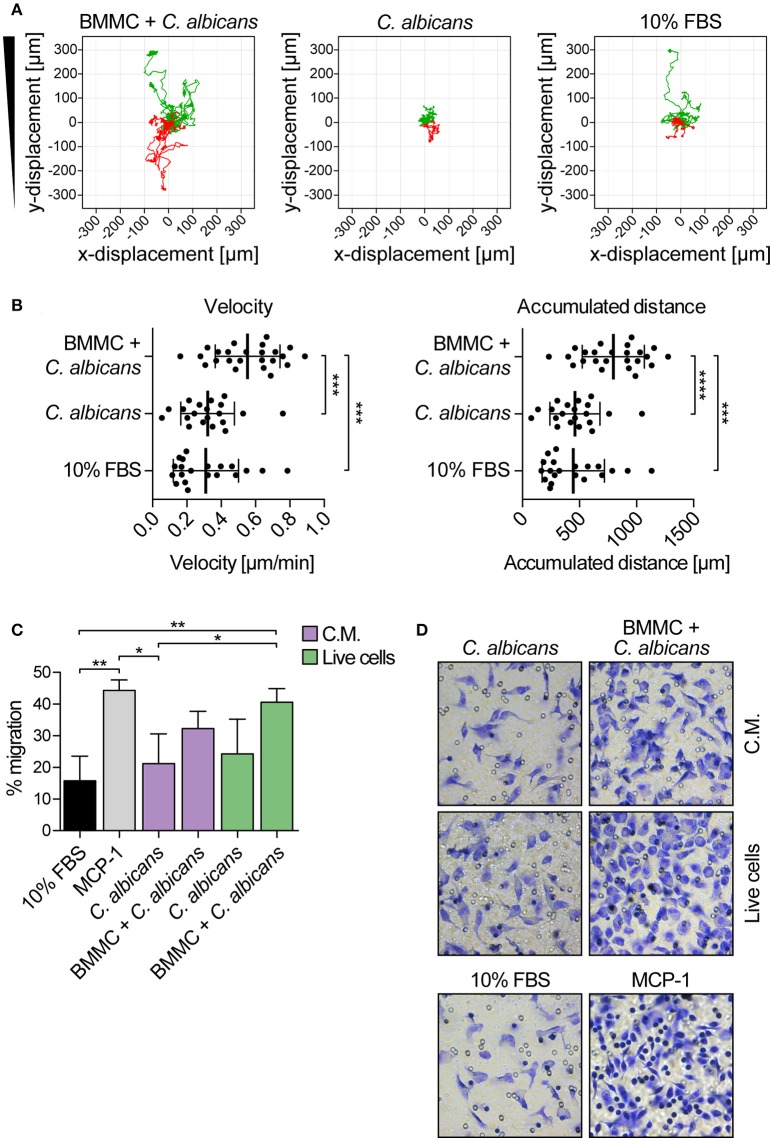
*Candida albicans-*infected MCs increase peritoneal macrophage crawling and promote their chemotaxis toward the infection. **(A)** Single cell tracks during 24 h chemotaxis experiment. Green tracks indicate single cells tracks moving toward the conditioned media, red tracks indicate cells moving away from the chemoattractant. Representative data of three independent experiments. **(B)** Albeit failing to attract macrophages, culture supernatants from BMMCs + *C. albicans* co-culture increased macrophages crawling ability. Data were analyzed with ordinary one-way ANOVA and Tukey's multiple comparison test. **(C)** Transwell migration assay was set up against a live *C. albicans-*MCs infection (live cells) or against the same conditioned media (C.M.). While the live infection induced a prominent migration of macrophages, conditioned media were able to induce macrophage chemotaxis only partially. MCP-1 was used as positive control of migration. Bars represent mean ± SD of three independent experiments. Data were analyzed with one-way ANOVA and Tukey's multiple comparison test. **(D)** Representative images of migrated macrophages, stained by crystal violet. ^*^p < 0.05, ^**^p < 0.01, ^***^p < 0.001, ^****^p < 0.0001.

The ability of MCs to induce macrophage chemotaxis was determined with the ibidi® μ-Slide Chemotaxis slides. For each experiment, peritoneal macrophages were purified from C57BL/6 mice peritoneal lavages and checked for F4/80, CD11b and MHC-II expression by flow cytometry and immunofluorescence (Figure 4). To determine the release of chemotactic factors during *C. albicans* infections, conditioned media of BMMCs- *C. albicans* co-cultures were collected after 3 h and used to assess migration. Conditioned media from living, germinating, *C. albicans* alone and complete RPMI media (10% FBS) were used as controls. Figure 5A shows all the single cells trajectories during 24 h incubation. Notably, conditioned media from BMMCs + *C. albicans* co-cultures induced a more evident movement of macrophages. Forward migration indexes (FMI) were calculated and showed no significant differences (FMI| = 0.0147 ± 0.0126 and FMI- = -0.0008 ± 0.0228) (28). Rayleigh test reported a *p*-value of 0.5327, thus indicating that cell endpoints were uniformly distributed. On the other side, macrophages incubated with BMMC + *C. albicans* culture supernatants moved with a higher velocity which resulted in greater accumulated distance compared to controls (Figure 5B). These data might indicate that, during *C. albicans* infections, BMMCs release soluble factors that increase macrophage crawling but do not promote their chemotaxis. On the other side, it is possible that the chemotactic factors might be unstable in the media. To further confirm this latter hypothesis, we evaluated macrophage chemotaxis toward a live *C. albicans* infection taking advantage of a transwell migration assay. Interestingly, conditioned media from infected MCs only partially induced macrophage chemotaxis but, on the contrary, live infection induced a prominent migration of macrophages (Figures 5C,D). Taken together, these data suggest that MCs can release short-lived soluble mediators which improve tissue-resident macrophage crawling and induce their migration toward *Candida* infections.

### Resting MCs partially inhibit macrophage phagocytosis ability

**Figure 6:**
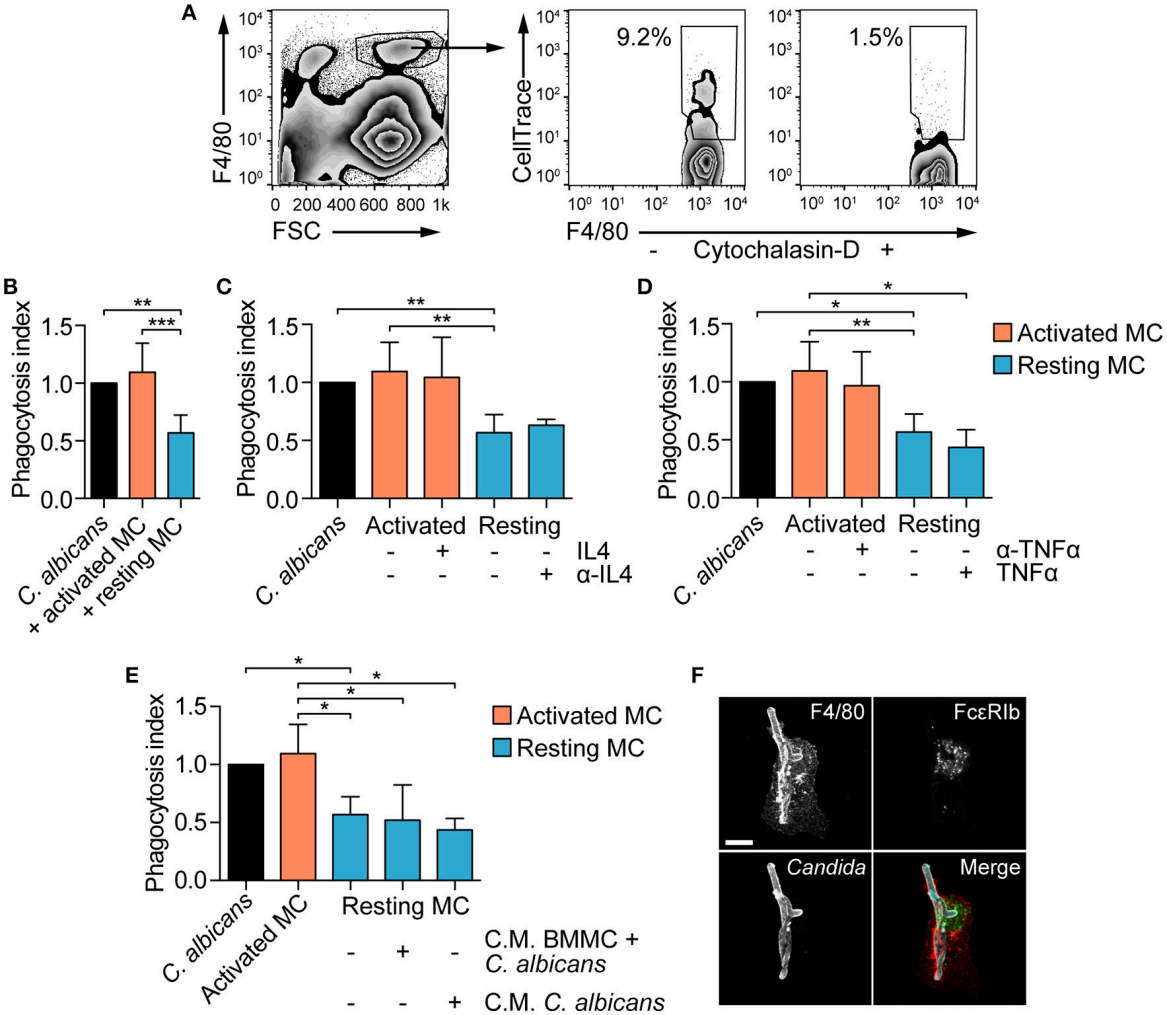
MCs activation influence fungal clearance by peritoneal macrophages. **(A)** Gating strategy used for the evaluation of macrophages phagocytosis. **(B)** *C. albicans* phagocytosis by macrophages is impaired by the presence of naïve (resting) MCs. The addition of exogenous IL-4 nor its neutralization with monoclonal antibodies **(C)** as well as the addition of TNF-α nor its neutralization **(D)** restored or inhibited macrophages phagocytosis. Phagocytosis was assessed by flow cytometry after 1 h of co-culture and the phagocytosis index expressed as the fold-change over the phagocytosis percentage of macrophages stimulated with *C. albicans* alone. **(E)** Similarly, the presence of conditioned media (C.M.) to resting MCs did not affect macrophages phagocytosis ability. Data were analyzed with Kruscal-Wallis and Dunn's multiple comparison tests. **(F)** Immunofluorescence analyses of macrophages-BMMCs-*C. albicans* co-cultures indicate that MCs and macrophages interact during the process of phagocytosis. Scale bar: 10 μm. ^*^p < 0.05, ^**^p < 0.01, ^***^p < 0.001.

MCs are known to modulate macrophages' phagocytosis ability (26, 29). To establish whether MCs phagocytosis of *C. albicans* could be responsible for a better fungal clearance by providing “eat-me” signals to tissue-resident macrophages, peritoneal macrophages were co-cultured with BMMCs and *C. albicans*, and their phagocytosis ability was determined. BMMCs were stimulated with CPD-stained *C. albicans* for 3 h, in order to allow phagocytosis of *Candida* germinated yeasts (from now on, these cells will be referred as “activated MCs”), scraped and seeded to peritoneal macrophages. Naïve BMMCs + CPD-stained *C. albicans* (referred as “resting MCs”) or CPD-stained *C. albicans* alone were seeded to peritoneal macrophages as a control. After 1 h of co-culture, the percentage of phagocytosis was determined by flow cytometry (Figure 6A). Albeit activated MCs had no effect on the phagocytosis of *C. albicans* by macrophages, resting MCs were able to inhibit macrophages phagocytosis (Figure 6B).

### Impaired phagocytosis of *candida* by macrophages is not dependent on MCs soluble mediators

MC-dependent inhibition of phagocytosis has already been described during bacterial infections and appear to be mediated by a quick release of IL-4 from MCs upon bacterial encounter (26). To determine whether naïve BMMCs inhibition of macrophages phagocytosis was dependent on quickly released IL-4, macrophages were stimulated in the presence of exogenous IL-4 or anti-IL-4 blocking antibody. Addition of recombinant IL-4 to activated MCs or the neutralization of IL-4 activity on resting MCs did not affect macrophages phagocytosis of *C. albicans*, suggesting that IL-4 is not involved in the modulation of phagocytosis (Figure 6C). Seen that *C. albicans*-stimulated MCs release TNF-α already after 3 hours (Figure 3B), we hypothesized that TNF-α might be responsible for the reversion of the phenotype by activated MCs. However, the addition of recombinant TNF-α to resting MCs or neutralizing TNF-α activity in activated MCs did not revert the phenotype (Figure 6D). To undoubtedly exclude a role of MC-derived soluble mediators in the modulation of macrophages phagocytosis, conditioned media were collected after 3 h of BMMCs and *C. albicans* co-culture or from *Candida* alone, and added to macrophages together with resting MCs and *C. albicans*. Again, phagocytosis inhibition was not reverted, suggesting that this mechanism is soluble mediators-independent but rather contact-dependent (Figure 6E). This hypothesis is further sustained by the fact that *in vitro* macrophages and BMMCs interact during *Candida* phagocytosis (Figure 6F). Intriguingly, activated MCs lost the inhibitory activity, indicating that fungal-dependent BMMCs activation can somehow down-regulate the expression of putative co-stimulatory molecules.

## Discussion

Every multicellular organism contains a rich and diverse microbiota and their interactions profoundly affect the fitness of both the host and the microbial community. The coexistence of these two entities is based on a fragile equilibrium between commensalism and pathogenesis which is maintained by proper mechanisms of activation and suppression of the immune system. Importantly, the disruption of this stable host-microbiota equilibrium can lead to pathological consequences (30, 31). A striking example is provided by numerous studies which reported a clear reduction in gut microbiota diversity on patients with autoimmune diseases (32). *C. albicans* is the most common member of the human and murine mycobiota and is found as a commensal especially in the colon and the vagina. When the equilibrium between the host and the fungus is perturbed (e.g., during broad-spectrum antibiotics treatment, or in conditions of pathological or pharmacologically-induced immunosuppression) *C. albicans* can overgrow and cause severe diseases as recurrent vulvovaginal candidiasis and invasive candidaemia (7, 10). The main effector cells involved in the control of fungal infections are neutrophils and macrophages but in recent years several studies reported that also mast cells might be involved in the outcome of pathological *Candida* overgrowth (12, 14–16). This should not be surprising as a growing body of evidence highlighted the concept that MCs are not mere effector of allergies and anaphylaxis but are rather involved in the maintenance of tissue homeostasis as well as in many pathological circumstances (3, 24). Accordingly, it was recently demonstrated that MCs play a pivotal role during *C. albicans* infections also *in vivo*, contributing to the inflammatory pathology occurring during initial infection but contributing to the control of fungal growth and dissemination, and to the activation of memory-protective Th1 responses upon re-infection (33). The present study provides novel proofs of the role of MCs as tissue-resident sentinels involved in the recognition of fungal infections and in a wider cross-talk with the commensal microbiota.

Previous studies demonstrated that MCs respond to fungal infections with *C. albicans* but often reported contradictory data. In order to provide additional elements of the interaction between MCs and *C. albicans* we set up an *in vitro* co-culture system using murine WT and dectin-1^−/−^ BMMCs. Time-lapse microscopy experiments showed that MCs tightly interacted with the fungus as soon as it switched to the hyphal form, in a way that resembled frustrated phagocytosis (21, 22). The formation of this phagocytic synapse was further characterized by immunofluorescence analyses which revealed that MCs were able to re-organize their α-tubulin cytoskeleton and to accumulate LAMP1^+^ vesicles at the interface with the hyphae. Interestingly, no differences were observed between WT and dectin-1^−/−^ BMMCs suggesting that other receptor than dectin-1 might be involved in the recognition of *C. albicans*. This finding is in line with the current belief that *C. albicans* is able to efficiently shield the β-glucan layer after the germination to the hyphal form, thus preventing its recognition by dectin-1 (34). LAMP1 accumulation at the frustrated phagosome was previously described in RAW246.7 macrophages spreading on IgG-ovalbumin micro-patterned surfaces and was found to be accompanied by the release of β-hexosaminidase (21). Very recently, it was also demonstrated that during the frustrated phagocytosis of *C. albicans* hyphae by RAW macrophages, LAMP1^+^ vesicles accumulated at the interface with the fungus and in close proximity of the actin cuff, suggesting that complete enclosing of the phagosome is not required for the recruitment of lysosomes to the phagocytic synapse (35).

LAMP1 is also considered a marker of degranulation in MCs but incubation of BMMCs with *C. albicans* even in the presence of serum (to allow the germination of hyphae) did not result in the release of β-hexosaminidase nor leukotrienes (36). A possible explanation is that granules' cargo was released directly on the fungal surface due to their close interaction and thus preventing their detection in the supernatants. It has been demonstrated that MC-derived β-hexosaminidase was able to disrupt *Staphylococcus epidermidis* cell wall, rendering mice more resistant to bacterial infections (37). Moreover, a similarly polarized degranulation was recently described and named “antibody-dependent degranulatory synapse.” Opsonized *Toxoplasma gondii* tachyzoites induced FcγR-triggering on MCs and the localized release of granule contents at the interface with the pathogen (38). On the contrary, we observed the release of TNF-α, IL-6, IL-13, and IL-4 during the co-cultures, especially during the stimulation with the yeasts, which was partly dependent on the recognition of the fungus by dectin-1. This observation might reflect the fact that MCs recognize the morphological switch from yeasts to hyphae, possibly by discriminating the composition of the outer layer of the fungal cell wall or through the recognition of cell wall debris released during the germination. It was demonstrated that the release of IL-4 during *C. albicans* infections *in vivo* was fundamental for the induction of a protective T_H_1 response during reinfection. As such, *il4*^−/^^−^ mice were more resistant than WT littermates during the first infection with *C. albicans* (probably due to the absence of a T_H_2 skewing) but failed to survive a secondary infection (39). The authors did not identify the source of IL-4 in this context but our data support the idea that MCs may account for the release of this cytokine.

Several reports demonstrated that MCs are able to phagocytose bacteria and fungi, and that in particular conditions they can also present antigens to autologous T cells (40–43). Nevertheless, their ability to kill phagocytosed pathogens is much more limited than “professional” phagocytes so we hypothesized that engulfment of *C. albicans* could be an early line of defense addressed to recruit tissue-resident macrophages and promote the clearance of the pathogen. Time-lapse chemotaxis experiments revealed that the soluble factors released by MCs during *Candida* infection failed to induce peritoneal macrophages chemotaxis but instead markedly improved their crawling ability. These results are in agreement with a previous study by Lopes et al. which reported that conditioned media from *C. albicans*-infected human MCs were able to recruit neutrophils but not circulating monocytes (17). Interestingly, the velocity of migration toward the pathogen was described to be important to enable a quicker clearance of *C. albicans* by PMN *in vitro* (44). This suggests that increased macrophage crawling might be important for the clearance of *C. albicans* also by increasing the probability of the encounter with the fungus. To further determine whether the lack of chemotaxis against infected-MCs conditioned media could be due to the short half-life of chemotactic compounds, we set-up a chemotaxis protocol against a live infection. Interestingly, live *C. albicans*-infected MCs induced a prominent migration of macrophages, compared to uninfected controls. Contrary to our previous experiments, we noticed a partial migration also against infected-MCs conditioned media. This could possiblty be the result of the increased cell crawling observed during live-imaging rather than proper chemotaxis.

To assess whether MCs engulfment could promote macrophage-mediated *C. albicans* clearance by providing “eat-me” signals (such as PtdSer residues or Calreticulin), co-cultures between BMMCs, peritoneal macrophages, and *C. albicans* were set up. Interestingly, we found that resting MCs were able to inhibit macrophages phagocytosis of the fungus. A similar phenomenon was described in a model of severe septic peritonitis, in which a very fast release of IL-4 by MCs upon bacterial encounter resulted in the inhibition of bacterial clearance by peritoneal macrophages (26). Nevertheless, the neutralization of extracellular IL-4, nor the stimulation with infected MCs conditioned media reversed macrophages phagocytosis of the fungus, suggesting that this phenomenon might rely on cell-cell contact. This should not be surprising since it was demonstrated that MCs modulated T and B cells activation through the OX40-OX40L and the CD40-CD40L axes, respectively (45–47). Although no studies on the interaction between MCs and macrophages which might provide mechanistic insight of this phenomenon are present to date in the literature, and although our experimental data do not directly prove this, it is tempting to speculate that MCs might constitutively express inhibitory molecules on their surface which inhibit tissue-resident macrophages in healthy conditions and thus promote tissue homeostasis. However, during infections these molecules could be rapidly downregulated, allowing a proper and rapid activation of macrophages.

Taken together, this data demonstrate that MCs can respond to fungal infections by tightly interacting with *C. albicans* hyphae and releasing pro-inflammatory mediators as TNF-α and IL-6. The fungal challenge also induced the release of the T_H_2 cytokines IL-4 and IL-13. While IL-4 has been correlated with a protective effect during fungal reinfection, IL-13 is known to promote intestinal goblet cell hyperplasia and increased mucin expression during parasitic helminth infections (48). Thus, it is possible that a similar mechanism might be involved in the elimination of *C. albicans* hyphae. Moreover, we demonstrated that MCs-derived soluble mediators can increase tissue-resident macrophage crawling and promote their migration toward the infection. Interestingly, resting MCs were found to limit macrophages phagocytosis of *C. albicans*: this result might reflect the ability of MCs to restrain effector functions of myeloid cells in homeostatic conditions, highlighting once more that these cells are important players in the maintenance of the equilibrium between the host and the microbiota.

## Author contributions

MDZ, BF, LR, and CP conceived and designed the experiments. MDZ, BF, GP, TZ, and GR performed the experiments. AA cultured the fungus and performed some experiments. MDZ and BF analyzed the data. MDZ, BF, and CP wrote the manuscript. All authors read and approved the final manuscript.

Research data were collected in cooperation with the employees of the Czech Centre for Phenogenomics supported by the Academy of Sciences of the Czech Republic RVO 68378050, and by the project of support program for large infrastructures for research, experimental development and innovation LM2015040 Czech Centre for Phenogenomics, and by the project of the National Program of Sustainability II LQ1604 both provided by Ministry of Education, Youth and Sports of the Czech Republic.

### Conflict of interest statement

The authors declare that the research was conducted in the absence of any commercial or financial relationships that could be construed as a potential conflict of interest.

## Supplementary material

Supplementary Video 1 MCs phagocytosis of *C. albicans* hyphae. Time-lapse microscopy showing BMMCs (stained in red) phagocytosing *C. albicans* hyphae during *in vitro* co-culture.
